# Anthropological overview of kangaroo care in community settings in Madagascar

**DOI:** 10.1186/s12905-023-02781-7

**Published:** 2023-11-23

**Authors:** Emilia Brazy-Nancy, Chiarella Mattern, Brigitte Irene Rakotonandrasana, Vonimboahangy Rachel Andrianarisoa, Patricia Norolalao, Azzah Al-Rashid

**Affiliations:** 1 Institut Pasteur de Madagascar, 1274 Ambatofotsikely Avaradoha, 101 Antananarivo, BP Madagascar; 2 Institut Pasteur de Madagascar, 1274 Ambatofotsikely Avaradoha, 101 Antananarivo, BP Madagascar; 3 Family Health Department of the Ministry of Public Health of Madagascar, Ministry of Public Health, 9 Printsy Ratsimamanga Ambohidahy, 101 Antananarivo, Madagascar; 4 Family Health Department of the Ministry of Public Health of Madagascar, Ministry of Public Health, 9 Printsy Ratsimamanga Ambohidahy, 101 Antananarivo, Madagascar; 5 USAID/Madagascar, Lot 207 A, Point Liberty-Andranoro Antehiroka -, 105 Antananarivo, Madagascar; 6 USAID/Madagascar, Lot 207 A , Point Liberty - Andranoro Antehiroka -, 105 Antananarivo, Madagascar

**Keywords:** Obstetric care, Kangaroo care, Preterm birth, Anthropology, Madagascar

## Abstract

The management of preterm births remains a major challenge in Madagascar, given the lack of equipped facilities in rural areas, and the absence of precise data concerning the incidence of such births. The World Health Organization (WHO) recommends the kangaroo method (skin-to-skin contact) for the management of preterm infants. In this article, we examine the conditions for success and the obstacles facing kangaroo care in community settings in Madagascar. We performed a qualitative research, collecting data from the districts of Antananarivo and Mahajanga in 2021. In total, 54 semi-structured interviews and two focus group sessions were conducted with the parents of preterm infants and healthcare professionals.

The obstacles identified by the research included incomplete and inaccurate knowledge, leading to heterogeneity in the practice of kangaroo care: arbitrary duration, lack of kangaroo care at night, exclusive breastfeeding, and only partially practiced skin-to-skin contact. We found that the conditions for success depended on: (1) the healthcare system, (2) local interpretations of pregnancy, and (3) the structure of the infant’s family. Failings of the medical system, cultural habits going against kangaroo care, and “nuclear” and/or “single parent” family structures undermine the application of this practice, jeopardizing upscaling efforts. We hope that the results of this research will guide community strategies for the management of preterm infants and kangaroo care.

**Supplementary Information:**

The online version contains supplementary material available at 10.1186/s12905-023-02781-7.

## Introduction

Preterm birth (before 37 weeks of gestation) is the leading cause of death in children under 5 years of age worldwide. Almost 1 million children die each year due to complications related to preterm birth, and many preterm infants suffer from psychomotor developmental delays and motor and intellectual disabilities [1]. Preterm birth rates are particularly high in low- and middle-income countries, notably in Southeast Asia and sub-Saharan Africa, which account for 60% of preterm births worldwide [2].

Over the last three decades, Kangaroo Care (KC) which can be defined as a method of care of preterm infants, has proved to be a safe and effective way to manage preterm and low birth weight (LBW) neonates, and to improve their survival. This is particularly true in low- and middle-income countries, notably those in which the necessary equipment and techniques for the management of preterm infants (e.g., incubators) are not available. The method was created in 1978 by two Colombian pediatricians who were inspired by the way female kangaroos carry their babies until they are mature. The central element of KC is the kangaroo position, which involves placing the baby in an upright position against the parent’s chest, in direct skin-to-skin contact. Exclusive breastfeeding is encouraged as the optimal choice for feeding. The third component of KC is outpatient care. This involves continuing the method at home, with the support of a reliable monitoring system [3, 4].

In 2020, the World Health Organization (WHO) published standards for improving the quality of care for clinically stable newborns with birth weights ≤2500 g [2]. It recommended kangaroo care for all newborns born preterm or with a low birth weight, as soon as possible after birth, with adequate support for the parents. According to WHO recommendations, the duration of skin-to-skin contact should increase gradually to become as continuous as possible, day and night; the only interruptions being for diaper changing. Kangaroo care can be given by mothers, fathers, or any other provider. Infants can leave the healthcare facility relatively quickly, if mothers receive appropriate support and follow-up at home. This type of care is generally provided until the infant reaches full term (a gestational age of around 40 weeks) or weighs about 2500 g.

Despite numerous efforts to implement this method, raise awareness and provide training, certain countries, including Madagascar, are finding it difficult to scale up KC practices. Following the introduction of KC practices by the United Nations Children’s Fund (UNICEF) in 1999, hospital professionals from Madagascar were able to receive training in Bogota, with the aim of applying these methods and transmitting them through cascade training to other healthcare personnel in Madagascar. In the Analamanga region, Compassion Madagascar, a national organization founded in 2010, is working to scale up KC at the community which means in Basic Health Centers (BHCs) and hospital levels. This association is working towards the establishment of KC centers and helping caregivers to master the method.

The implementation of KC methods has been developed in various ways. The Public Health of Ministry of Madagascar has included this method in the training of professional healthcare personnel through the curriculum in maternal and neonatal health (MNH) and the training curriculum of the Malagasy Society of Pediatrics (SOMAPED) at hospitals in the regions of Analamanga, Boeny, and Atsinanana. About 15 KC units have been set up in various maternity units on the island and in two neonatology departments (Mahajanga and Antananarivo). A study conducted in 2018 within a KC unit [5] in the capital highlighted the importance of analyzing the feasibility of scaling up this method in the community setting^1^ (in BHCs), which was the purpose of this study.

The objective of the qualitative research, conducted in two areas in Madagascar, was to analyze the conditions favoring the success of KC and the barriers potentially hindering the introduction of this approach at the community level, in basic health centers.

We hope that the results of this study will guide community strategies for the management of preterm infants by KC.

Finally, throughout this article, we have preferred to use the term “kangaroo care” suggested by Nyondo-Mipando et al. [6] rather than “kangaroo mother care” to raise awareness of the excessive responsibility loaded onto mothers, and because this term underestimates the important roles of the other members of the family, including the father.

## Methods

This article is based on the results of 18 months of anthropological research conducted in 2021 and 2022 in two districts of Madagascar: Antananarivo (the capital city located in the center of the island) and Mahajanga (a port city in the northwest of the island). The ‘Prema’ study was conducted by the Health & Social Sciences team of the Pasteur Institute of Madagascar in collaboration with the Ministry of Health (Direction Santé Familiale) and it was funded by the U.S. Agency for International Development (USAID) Madagascar under the RISE (Research, Innovation, Monitoring, and Evaluation) project.

### Zones of study

The regions were selected with the major research partner (Ministry of Health) according to the following two criteria: (1) the proportion of low birth weight newborns (high in Analamanga, where Antananarivo is located, and lower in Boeny, the region in which Mahajanga is located).^2^ This criteria has been chosen in the absence of data on preterm birth infants, and (2) the practice of KC (widespread in Analamanga and less practiced in Boeny). BHCs practicing KC were identified in these two regions. The study was then conducted with the BHCs at which KC was practiced: two in Antananarivo and five in Mahajanga.

### Qualitative research participants and recruitment

The study targeted two categories of people: (i) those involved in the KC of a preterm child born in the 6 months preceding the research or those who were in the process of performing it, i.e., the parents or other family members participating in KC, and (ii) the health personnel of the seven selected BHCs. In theory, the BHCs registers record premature births in the health center, making it possible to clearly identify which parents have been involved with the practice of MK. The health center managers facilitated the identification of the mothers targeted and the meeting with them. In a second phase, the family members involved in the practice of MMK were contacted to take part in the research.

### Type of data collected and entered

For this study, we planned to conduct a minimum of 50 semi-structured interviews with 30 mothers of preterm children and/or family members practicing KC (15 per zone) and 20 biomedical caregivers (doctors, midwives, nurses) and traditional practitioners involved in the KC (10 per zone) Additional file 1. Additionally, we planned to conduct 2 focus-groups: 1 focus group per area of which each focus will include 5 mothers and/or people who have practiced KC.

The interviews are conducted in Malagasy. They are recorded and then transcribed in their entirety into a Word file. The interviews are then translated, in their entirety, into French.

### Analysis

This research responds to a need expressed by the Ministry of Public Health in Madagascar to test the possibility of scaling up the method in a community setting. The themes addressed are therefore intended to meet this need.

A thematic analysis was used for data processing. The interviews were coded according to a list of codes established before data collection, with a code being the label used to mark a concept (for example: the care path during pregnancy). Coding was conducted by the first author of this article. Coding is the first step in the analysis of qualitative data. A cross-sectional thematic analysis was performed on the translated data by filling in an analysis grid. Two analysis grids were designed for this study: one for the interviews with the mothers of preterm infants and the other for the interviews with healthcare professionals. The grids covered data corresponding to several themes: the management of preterm infants at the BHC, the transmission of information by healthcare professionals, the perception of KC social norms and family involvement in the practice of the method at home, expectations in terms of the efficacy of KC, and expectations for improving KC. With this approach, we were able to highlight recurrent and divergent elements between the interviews for each theme, making it possible to highlight the main trends, while considering the particular features of the life histories of the interviewees, and their representations.

For FGs, we focused on themes that emerged from interviews. Notably the information received about MK by the nursing staff, the practice of MK in terms of duration, practice of MK during the night and other community case management of prematurity.

### Ethical considerations

The Ethics and Biomedical Research Committee of the Ministry of Public Health declared this study to be exempt from review and authorized data collection. All interviews were conducted with written informed consent from the interviewees. An information note was read to the interviewees and explained to them in detail. The importance of the study and the confidentiality of the interview were stressed during this explanation. After receiving all the necessary explanations concerning the study, participants were asked to sign a consent form indicating that they agreed to be interviewed.

### Knowledge transfer

As a means of maximizing the impact of this study, knowledge transfer activities^3^ were established within the framework of this project during and after data collection, with restitution of the results to the project partners and all potential users (various departments of the Ministry of Health, international organizations, healthcare professionals) and the joint formulation of recommendations based on these results. This approach ensured that the recommendations were both achievable (considering the resources available, the context, etc.) and relevant. The research team worked with the Renard group from the University of Montreal on these recommendations, which are listed at the end of this article.

## Results

In total, 54 interviews were conducted, including 32 with mothers of preterm infants and/or family members practicing KC, and 22 with healthcare professionals (3 doctors, 16 midwives, 3 nurses) involved in KC. We conducted two focus groups (FG) with other parents of preterm infants, focusing on the themes that emerged from the semi-structured interviews, to examine the consistency of the responses given. Those practicing KC were interviewed at home, whereas care staff were interviewed at the BHCs. The interviews were conducted in Malagasy.

### Characteristics of the targeted population and description of the BHCs

The results cover the social and demographic characteristics of the 32 individuals involved in the KC of premature infants interviewed (Table 1). One of these individuals was the father of the infant, but all the others were mothers, with, on average, two children each. The mean age of the mothers was 26 years. Most of the interviewed (29/32) worked in the informal sector (housekeepers, laundry workers, saleswomen). Only 3/31 mothers had finished secondary school (obtaining a baccalaureate).

**Table 1:** Social and demographic characteristics of the 32 individuals involved in the KC of premature infants interviewed

	Zone		Total
	Antananarivo	Mahajanga	
Gender			
Female	16	15	31
Male	0	1	1
Age			
< 20	4	6	10
20–30	9	8	17
30–40	3	2	5
> 40	0	0	0
Educational level			
No level	3	8	11
Primary and above	7	6	13
Secondary and above	6	2	8
Number of children			
< 2	4	4	8
=2	6	7	13
> 2	6	5	11

Most of the mothers family model is cohabitation outside marriage (*fisehoana* or *vodi-ondry*). The family structures were single-parent families, which was the case for 20/31 mothers (mostly single mothers), nuclear families (couples living under the same roof) for 8/31 mothers, or extended families (4/32). The family context of the women interviewed was largely affected by violence and/or breakdowns of relationships due to violence. Following break-ups, the preterm children become the responsibility of the mother (and her family if there is an extended family).

The healthcare professionals interviewed were mostly midwives (3 doctors, 16 midwives, 3 nurses) and their mean age was 35 years.

The two BHCs in Antananarivo have a special KC room decorated with paintings illustrating mothers carrying their infants in *lambaoany* (traditional fabrics) and posters providing information about the positioning of the baby and the advantages of KC. However, the two rooms are used by all users of the service and the hygiene infrastructure is unusable. In Mahajanga, none of the five BHCs has a KC space. The rooms allocated for the postpartum period are overcrowded because they lack sufficient modern equipment (beds, etc.).

### Identification and management of preterm infants nationwide

#### Difficulties identifying cases of preterm birth and determining its impact on therapeutic orientation

One of the major challenges faced by healthcare professionals in the management of preterm infants is the identification of the infants concerned. In the health centers investigated, preterm infants were essentially identified based on the appearance of the baby at birth, and particularly low birth weight. It should be possible for professionals to identify preterm infants by observing intrauterine growth retardation with an ultrasound device, but no such devices are available at any of the BHCs studied. The other element that can help to identify preterm infants is the precise determination of the gestational age, also by ultrasound methods. The healthcare professionals interviewed deplored the women’s lack of knowledge about the date of their last menstrual period.

*“When the child is born before 37 weeks of gestation, it is preterm, but if we look at the length of the pregnancy, the date of the last menstrual period is not precise because the mothers do not remember it. So, we don’t rely too heavily on the length of pregnancy, but more on the weight. It’s more reliable.” –* Antananarivo.

In light of this difficulty and to ensure appropriate care despite the lack of information about the term at which the infant is born, healthcare professionals use the criterion of “low birth weight” to guide management. None of the registers observed during the investigation mentioned a “preterm” child. We understand that preterm birth is not accurately reported at the national level by the Ministry of Health, which may explain the lack of precise statistics. Therefore, as the child is not officially recognized as “preterm”, there is no communication between the healthcare professional and the parents concerning the diagnosis of “prematurity” for their child. As we will see below, parents know little about the term “preterm” and very few use it to characterize their child. Health professionals deem newborns eligible for KC meeting the following criteria: low birth weight, i.e., a weight of less than 2500 g, with a sucking reflex and not suffering from respiratory distress syndrome or other serious illnesses.

#### Implementation of KC for “low birth weight” children

Our interviews with mothers indicated that they did not know about KC before the birth of their child. Only one of the 32 people involved in KC interviewed reported prior knowledge obtained through a radio spot. The BHC is the leading source of information on low birth weight and KC.

If there are no contraindications (respiratory problems, absent sucking reflex, etc.), healthcare professionals teach the mothers and other caregivers present how to practice KC. In Antananarivo, slings (single-color elastic fabrics) that facilitate the carrying of the child are loaned to mothers by Compassion Madagascar. In the Mahajanga region, mothers carry their babies in traditional fabrics. In both regions, healthcare professionals suggest that families buy diapers for infants from grocery stores or businesses close to the site at which the baby was delivered, rather than using the washable cloth nappies generally used by mothers. They justify this request both by the fact that it limits the hygiene problems that can be caused by unclean nappies and by the desire to optimize the time for which the infant is carried. They also claim that commercial diapers have a greater absorption capacity, making it possible to limit the interruption of the practice of KC and to keep the child warm for longer. As indicated by the following interview excerpt, the health professionals interviewed are aware of the financial obstacle that this represents:

*“There are people who cannot buy diapers and we try to convince them by telling them that the baby must be kept warm, because it would be difficult to return this heat to the baby if the baby is cold. Cloth diapers have to be changed frequently if the baby “pees” too often, which is why the baby should be put in a diaper.”* Midwife, 29 years of age, married, one child.

In addition to these initial difficulties, the length of the postpartum stay at the health center is also a constraint on the management of low birth weight infants mentioned by healthcare professionals. Indeed, none of the mothers interviewed stayed for more than 24 hours at the BHC after delivery, despite the encouragement of healthcare professionals to stay for 3 days in the room allocated to postpartum care. There were multiple reasons for this rapid departure from the BHC: lack of privacy, hygiene, and accommodation for those accompanying the mother, and too long an absence from home.

Nevertheless, the mother and her newborn can go home once the infant is feeding well, the infant’s body temperature remains stable in the “kangaroo” position, and the infant is gaining weight. As most infants are still preterm when discharged from the facility, mothers are made aware of the importance of maintaining KC at home. The information transmitted mostly concerns the importance of maintaining continuous skin-to-skin contact. Little information is provided about the time for which the infant should be carried or physical aspects of the child that might indicate a change in growth. The healthcare professionals justified this in terms of the need to simplify the information given to mothers, as indicated by the following interview excerpt:

*“As the gestational age is not very well-defined, it is difficult to give a number of weeks for which KC should be performed, so we say to do it until the child gains weight.”* BHC Midwife – Antananarivo.

The mothers deplored the lack of clarity and content of the information received on this subject. The information received mostly concerned the possible cessation of KC when “*we see that the child is gaining weight*”, as indicated by a young mother in Antananarivo. The mothers considered this information to be too imprecise, and sometimes stopped KC after only a few days.

KC, as promoted by the WHO, requires regular follow-up by a qualified professional living near the mother’s house. These regular home visits (HVs) ensure that the techniques taught by the healthcare professionals at the BHC are implemented. None of the healthcare professionals interviewed said that they carried out HVs, justifying this by the lack of human resources at their health facility, and a lack of means to reach the mothers, who sometimes live very far from their center. HVs are not part of the designated tasks of healthcare professionals in public BHCs.

Healthcare professionals try to circumvent this difficulty by inviting mothers to visit the BHC 1 week after childbirth, with the purpose of following the change in the infant’s weight, performing the first vaccinations, and monitoring care (sutures for cesarean section or episiotomy, breastfeeding follow-up, etc.). Concerning this subject, a midwife from Mahajanga stated that:

*“Each time she does the vaccination, we can see if the infant is growing well…”* 38 years of age, married, two children – Majunga.

Healthcare professionals also recommend working with community agents, both for follow-up and to convince mothers that they should go to the health center in the event of a problem.

Nevertheless, this leads to many women being lost to follow-up, raising questions among healthcare professionals concerning the practice of KC at home:

*“We don’t know if they really do it or they really don’t! So that’s the difficulty.”* Midwife, 35 years of age, married, five children.

Healthcare professionals assume that mothers stop the practice once at home:

*“When they leave here, they don’t practice it at their homes.”* BHC Midwife, 31 years of age, no children– Antananarivo.

Some mothers lost to follow-up are then contacted by telephone on the personal initiative of certain health professionals.

#### The economic burden of referring underweight children

Infants not considered fit for KC with a birthweight below 1 kg are referred to the nearest hospital, which should normally be equipped with incubators. According to healthcare professionals, families fear referral to a hospital, notably due to the high cost of hospitalization and the direct and indirect costs associated with hospitalization.

Healthcare professionals affirm that families that do not want to go to the hospital ask for KC:

*“We should send them to Androva [hospital] to take care of the child, but often when the family sees that the child is robust then they say: no, we are not going there, and when they don’t go, we show them the kangaroo technique. We teach it to them while they’re still here”.* BHC Midwife, 34 years old, married, two children – Majunga.

Healthcare professionals therefore offer this solution as a last resort while stating that KC cannot replace special care.^4^

Three conditions for the success of KC emerged from the interviews and focus groups with the participants. These conditions favor the success of this approach and relate to both the health facilities in which mothers give birth and their homes.

#### Brakes and levers relating to training and infrastructure

These conditions for success concern the training in KC provided to healthcare professionals and then to the characteristics of the infrastructures and equipment available to mothers and healthcare professionals for the practice of KC, both within healthcare facilities and at home.

### Quality of KC training provided to healthcare professionals

In terms of the training of healthcare personnel in KC in the BHCs, there is not yet a national policy for the management of preterm infants. KC is taught in hospitals, but not systematically at all levels of the healthcare pyramid. Nine of the 22 healthcare professionals interviewed reported having received/read a module on KC during their medical training, seven indicated that they had received training during the exercise of their profession (from Compassion Madagascar, for example), and five complained that they had not received any training. Preterm infants should normally be managed at referral hospitals. Training for healthcare professionals in BHCs is essentially based on the Ministry of Health’s curriculum and focuses on the management of low birth weight newborns, preterm labor, and the KC technique. More specialized content is currently being developed for the KC module.

This study highlights heterogeneity in the level of training and in the providers of training, mostly civil society actors in Madagascar or international institutions. For example, we observed a much higher level of training among the healthcare professionals interviewed in Antananarivo, due to the presence of Compassion Madagascar, with six healthcare professionals trained in KC in Antananarivo but only one in Mahajanga. In Mahajanga, training is provided at hospital level. Cooperation agencies, such as the Japanese International Cooperation Agency (JICA), helped to introduce KC into the hospital environment in 2010. However, there are no initiatives specifically targeting health facilities in the community, such as BHCs. Mahajanga health professionals are therefore much less equipped to teach the method due to their own lack of training, as attested by this midwife:

*“I have not yet had any training. I gained some knowledge during my internship at the university hospital.”* 34 years old, married, one child - Majunga.

In some cases, the only information available about KC is the information transmitted informally during exchanges between healthcare professionals or during initial training.

*“I never had training specifically on KC, but we talked about it from time to time during training for maternal and newborn care.”* Midwife, 42 years old, married, two children - Majunga.

As these excerpts suggest, KC is delivered without standardized guidelines, with content and formats that vary depending on who is responsible for training.

Many healthcare professionals try to overcome the lack of information and specific training on KC by reading documents available at the BHC. Healthcare professionals described having used manuals and/or informal exchanges between peers when a preterm child was delivered. This was the case, for example, for a midwife in Mahajanga, who was officially trained in KC through the provision of a module in Emergency Obstetric and Neonatal Care (SONU) set up by the Ministry of Health. After receiving this training, she in turn trained her team of midwives and volunteers.

The advice given on the duration of KC, skin-to-skin contact, and the application of KC during the night lacks clarity and precision, as shown by the information received by this mother in Antananarivo: *“the midwife did not say much other than take off his clothes and place him on your body”.* The knowledge of healthcare professionals about KC is, therefore, heterogeneous and this, in turn, affects the quality of the information delivered to families, as discussed below. In addition to training, our interviews revealed that the characteristics of the infrastructures in which the management of LBW infants by KC takes place can also promote or hinder the correct application of the method.

#### Infrastructures

Two key characteristics relating to infrastructure are considered here: the lack of space dedicated to KC training and the lack of accommodation for visitors.

First, learning KC at the BHC is favored by having a dedicated space, guaranteeing the privacy that parents and families expect. Compassion Madagascar has set up spaces specifically dedicated to KC in Analamanga, but we found that these spaces were occupied indiscriminately by all BHC patients, not specifically the mothers of preterm infants. However, the practice of KC requires skin-to-skin contact with the child and the person carrying the child. According to our research these are mostly women. Who must at least partially expose their body. According to healthcare professionals and mothers, without the appropriate environment that allow for intimacy and calmness, KC cannot be adopted and practiced effectively.

*“It would be nice to put a screen here, but we can’t afford it! We need to separate out those who are not doing kangaroo care! But that is not possible, and in the end, we put everyone who gives birth together in this room, even those who do kangaroo care!”* BHC Midwife, 48 years old, four children – Antananarivo.

In addition to the elements described above, this lack of a dedicated space contributes to the early departure of mothers from BHCs.

Second, the provision of accommodation for accompanying individuals at the BHCs is a central element that would promote the implementation of KC. The presence of other people willing to carry the baby makes it possible to alternate carrying duties, allowing the mother to rest. Those accompanying the mothers are treated like care assistants, given the lack of water, hygiene facilities, and recurrent drug shortages in the BHCs. They help to feed the mother and take care of her during her stay, functions that are essential to promote longer stays at the BHCs at a time considered important for the practice of KC.

#### When material becomes indispensable

In theory, it is not necessary to have a particular type of sling for KC. Based on official WHO recommendations, any scarf or fabric can be used to carry the infant. However, the national association lends mothers slings for a limited period at the BHCs in Antananarivo. Families therefore consider these slings to be essential, as they provide a feeling of security, but this can limit the continuation of KC if this material is no longer provided or no longer available. Without the sling provided by the BHC, some mothers feel that they are not practicing the method properly at home. This may lead them to stop applying the method once the sling loaned by the BHC is returned, or to use other techniques (see below):

*“We must keep the child warm. It was important to use the bottle for the child, because we had to return the sling.”* Mother***,*** 21 years old, one child – Antananarivo.

This situation was also noted by healthcare professionals, as illustrated by this verbatim statement:

*“We put the baby in a sling, but then, if there isn’t one, it becomes a reason for them not to do it.”* Midwife, 35 years old, married, five children - Majunga.

In addition to these “levers” that health professionals and the individuals practicing KC described as facilitating the practice of KC, our results also indicate elements more closely related to local notions concerning pregnancy and the birth of a child.

#### Local context: an aid or obstacle to KC?

Among the many elements linked to local contexts in Madagascar, we selected three themes relating to local ideas about pregnancy, family support, and gender.

#### Local practices concerning pregnancy and the postnatal period

Beliefs about pregnancy and the postnatal period may aid the practice of KC, particularly as techniques for managing preterm or LBW infants may be consistent with KC. Beliefs may also hinder the application of KC (the bottle technique) because they replace KC or jeopardize its implementation.

#### The bottle technique

In extended families (related group of several people living in the same household), practices in the care of newborns are influenced by older women in several regions of Madagascar. In this study, we observed that practices were strongly influenced by elders, which can hinder the acceptance of KC, as deplored by this midwife: *“It is especially the grandmothers or the elderly who are stubborn. They are the ones who are still difficult to convince [to perform KC]. They pretend to accept it when they are at the BHC.”* 40 years old, two children – Antananarivo***.***

As this excerpt indicates, caregivers perceive the role of elders and their support as being determinant in the practice of KC.

Many elders advocate for the bottle technique to care for a child considered “too small” or “too light” at home This technique involves surrounding infants with hot water bottles and wrapping them in a blanket to keep them warm. The bottle technique, which is now strongly discouraged by the WHO, is still widely used. The technique was observed at our two study sites and was unanimously adopted in Antananarivo, where temperatures are lower. Some healthcare professionals themselves advised the use of these “grandmother’s” techniques because they are consistent with the need to keep the child warm.

*“I met a midwife who was a bit old here. At that time, there was not much training, but they [healthcare professionals] practiced the bottle technique with hot water … they wrap the bottle with something and put it right next to the baby, I remember at the time they said to me that: ha! It is really very effective for children who are underweight! It was the midwives who did it!”* Chief doctor, 55 years old -Mahajanga.

This technique is used by families in alternation with KC to compensate for the impossibility of maintaining KC continuously. It is used when the mother is doing household chores, goes to work, or if the mother or family thinks that it is not possible to practice KC at night.

#### Mifana/confinement and thermal protection

In the local culture, it is considered important to keep newborns warm, as indicated by the custom of placing hot water bottles near infants and wrapping them in blankets, placing them in direct sunlight in the winter months to warm up, and administering massages with zebu fat or honey, which is supposed to be warming. *Mifana*, a custom of postpartum confinement specific to Madagascar and practiced in both Mahajanga and Antananarivo, allows the mother to nurse her infant for a month without doing household chores. This practice is favorable for the adoption of KC by the mother, unless she is alone. This custom is advantageous in that it prohibits anything that is “cold” and encourages the mother and infant to stay warm. Nursing mothers limit their exposure to cold water for fear of transmitting the coldness to the baby through their breast milk. The interviewees were aware that cold is a risk for newborns, and this may be beneficial for the implementation of KC. However, in some cases, this injunction to keep the child warm was seen as contradictory to the need for the child to be naked, in skin-to-skin contact with the parent. During our observations, we noted that many families practiced the recommended posture, but with the child in a bodysuit, as described by this caregiver: *“they put on his clothes and it’s after that they practice this [KC]”*, which is contrary to one of the first conditions of KC: skin-to-skin contact. The nudity of the child is considered to be a risk, so mothers dress their infants before placing them against themselves in the usual position for KC.

#### Carrying and breastfeeding

Mothers who are used to carrying their child on their back sometimes find it strange to carry their baby on the front:

*“Here, people are used to carrying children on their backs but here we do the opposite. We do not carry the child on the back but put it against our chest and we must attach the child.”* Midwife, 32 years old, married, two children - Majunga.

Nevertheless, this element is not considered an obstacle in the adoption of KC according to healthcare professionals and parents, who adopt the new position without difficulty when the benefits for the child are well understood.

None of the mothers exclusively breastfed in accordance with the official recommendations, throughout the period of KC. All the mothers mentioned the importance of exclusive breastfeeding, but the extent to which this is put into practice depends on the sociocultural context of the individual. For example, the practice of *mifana*, a period during which breastfeeding is facilitated, allows compliance with this recommendation. However, even during this period of rest, breastfeeding is combined with the use of rice broth and/or Nursie (a brand of formula milk): *“I give him a lot of rice broth so that he is robust!”* Mother, 35 years old, three children – Antananarivo. In addition, the introduction of complementary foods is used as a strategy to compensate for the absence of the mother, who has household responsibilities to fulfill. We observed this practice during our discussions with the mothers and a midwife also noted: *“They give something other [than milk] to the child.”* 36 years old, married, two children - Mahajanga. One caregiver said that she prescribed Nursie for preterm infants without a sucking reflex, which highlights the level of ignorance of providers about the practice:

*“When the baby is unable to suckle, the mother is asked to buy a pre-Nursie if she can afford it.”* Midwife, 36 years old, married, two children - Mahajanga.

#### Family support and type of family structure

“Family support” refers to the help provided by other people (other members of the family or the community) to achieve KC. We observed that KC practices were performed more diligently by extended families (or with help from the community), due to the additional support available. This social mechanism makes it possible to reunite favorable conditions for KC. For example, by performing household chores or essentially everyday tasks, allowing the mother to allocate time to carrying the baby. Replacing the mother for KC also ensures the continuity of skin-to-skin contact.

Continual skin-to-skin contact is more difficult if the mother lives in a nuclear family (without an extended family to allow a better distribution of domestic tasks), particularly, if the husband is absent. Most mothers interviewed were single, which made it difficult for them to practice *mifana* and continual skin-to-skin contact due to the need to perform household chores, such as cooking, cleaning the house, and washing clothes. For example, mothers said that they were unable to cook during skin-to-skin contact with the child. Carrying a child around also gets in the way of household chores. If a woman does the housework in the morning, she can only carry the child in the afternoon, once she has finished her work or household chores. Mothers complained about this inability to continue KC:

*“It has become like a great burden for me alone”* Woman, 27 years old, three children – Antananarivo.

The healthcare professionals are very aware of this situation, as illustrated by this statement:

*“If we don’t help her [the mother], it is possible that the KC will be interrupted, because it is tiring if it is only the mother who does it”.* Midwife, 42 years old, married, two children – Majunga.

#### Gender roles

Fathers play a small role in the care of children and are sometimes not very engaged in society itself: chores and childcare are mostly assigned to women. These gender roles influence the sensitization of healthcare professionals to families, leading them to pay more attention to the grandmothers and aunts of the infants who accompany the mothers. Healthcare professionals say that they cannot involve fathers because they are rarely present during deliveries: *“With us at the BHC, there are always people accompanying the mother during childbirth, but they are rarely fathers, rather the mother, the mother-in-law...”* BHC Midwife – Antananarivo.

Only one mother said that her husband helped to carry the child during KC. This was confirmed during the interview by the father, who said that he helped by carrying his child while his wife was working to support them. These exceptions show that it is possible for male figures to participate in KC, despite the cultural assignment of female and male roles making it difficult for fathers to participate in this practice.

#### Perception of the vulnerability of the child

Other conditions may or may not favor skin-to-skin contact with the infant, such as the mother’s view of her child’s vulnerability. Skin-to-skin contact can be perceived as uncomfortable for the infant, with the infant considered too fragile to be held in this position, significantly decreasing the chances of this practice being implemented: “*Because the child is still very small and they are afraid to do it”* BHC midwife, 31 years old, no children– Antananarivo. Mothers also said that they were afraid to implement KC at night, for fear of crushing the child. None of the women interviewed performed KC at night. The infant was instead generally placed on a bed next to the mother and wrapped in blankets. The bottle technique was also used overnight for preterm infants.

## Discussion

This study reveals a general willingness of mothers to practice KC and to implement the practice of skin-to-skin contact with the use of *lambaoany* or slings lent by BHCs. Nevertheless, levers can also become obstacles, depending on the context, favoring incomplete and imprecise knowledge and leading to discrepancies in the implementation of KC (arbitrary duration of KC, abandonment of the practice at night, and misunderstanding of the need for exclusive breastfeeding and skin-to-skin contact). We identified three themes with a direct impact on the practice of KC: (1) the healthcare system, (2) local ideas concerning pregnancy, and (3) family structure. These themes intersect with many barriers to the practice of KC in the community already reported.

We noted a lack of institutional support for the implementation of KC at community level, as evidenced by the absence of a national curriculum for healthcare professionals. This contrasts with the situation in other countries, such as Brazil, Colombia, Ghana, Indonesia, and Uganda, in which national guidelines for the implementation of the practice have been established, [7, 8]. Real data concerning preterm births were also lacking, the only indicator available being birth weight.

This lack of institutional support is accompanied by observable deficiencies in the healthcare system. The absence of adequate infrastructures presents an obstacle to providing the intimacy and hygiene for this practice considered essential in Madagascar, as elsewhere [7, 9–11]. Adequate training for healthcare professionals and follow-up at home is considered an essential component by the WHO but is lacking in Madagascar and other countries with similar structural conditions [12–14].

The number of trained healthcare professionals was higher in Analamanga, but we nevertheless found that most of the healthcare professionals interviewed were untrained and were teaching KC without standardized guidelines. This challenge has also been observed in other countries, including Ghana, Pakistan, India, and Uganda [8, 15, 16]. However, these healthcare workers play an important role in educating families, because the information they transmit influences the knowledge and practices of families in terms of preterm infant management [7, 17]. The lack of knowledge about KC among healthcare professionals may have a negative impact on the implementation and acceptance of the method by families (Seidman et al., 2015). Mothers were aware of the benefits of KC for their infants, but they were not aware of the possibility of preterm birth or KC before the delivery, leaving them unprepared and with limited coping skills. The education of families before the delivery is, therefore, considered crucial [7, 10, 18], because raising awareness at the prenatal stage increases the likelihood that KC will be accepted when the time comes [14]. Compassion Madagascar, which is working to scale up KC in the country, therefore has an essential role to play in compensating for the lack of training and infrastructure in the regions of Analamanga, Itasy, and Vakinakaratra. However, this association cannot meet all the needs of the country on its own.

Historically, child healthcare interventions have focused on the mother-child relationship, without taking into account social structures and cultural systems that might influence health-related beliefs and behaviors [19]. In extended families, older women greatly influence care practices for newborns in several regions of Madagascar [20], as already observed in Senegal and Mauritania [19, 21]. The mothers interviewed expressed a sense of value in maintaining traditional methods of newborn care, as also observed elsewhere [16, 22], especially recourse to a birthing assistant or “matrone” (*fakabe*) and techniques transmitted by grandmothers (the bottle technique). The custom of keeping infants warm and protecting them from the cold can positively (maintaining warmth) or negatively influence the practice of KC, as many families think that the child must be dressed to conserve heat, which greatly limits skin-to-skin contact. We found that the use of these methods was accompanied by a partial practice of KC, highlighting the desire to maintain a balance between these traditions and KC, perceived as a biomedical innovation emanating from BHCs.

Our results show that family structure strongly influences KC practices. Study participants indicated that support from their mothers, aunts, sisters, and husbands during the first few days after childbirth facilitated KC and/or was expected. The extended family provided favorable conditions for KC at home. A lack of family support was a clear obstacle to the successful practice of KC, as previously documented in the hospital setting in Madagascar [5] and highlighted by a large number of studies showing the importance of collective family responsibility [9, 16, 22–24] p10. Most of the mothers interviewed were single mothers and did not, therefore, benefit from such favorable conditions, raising questions of vulnerability at two levels. These women were at risk of preterm birth due to the absence of the father or the conjugal violence they suffered (both social risk factors for prematurity). For the same reasons, they were also at risk of not having the optimal social conditions to apply KC. For mothers in nuclear family structures (cohabiting with the fathers), almost none of the fathers carried their child for KC, due to the cultural assignment of feminine and masculine roles in childcare. Many authors deplore the absence of the father and highlight the importance of including fathers in the care of their children [22, 25–27]. Lydon et al. showed that, in Malawi, men are far from opposed to participating in the care of their preterm child but are constrained by societal gender roles [25]. Our study highlights a few rare exceptions, showing that it is possible for male figures to participate in carrying the child, such as the father we interviewed. These individuals can be seen as engaged in reform (i.e., out of step with the dominant behaviors), making it possible to drive changes, because they diverge from the function assigned to them [28]. It has been shown that there are fewer barriers to the practice of KC in societies with more equal gender roles (e.g. Scandinavian countries). In Brazil, paternal involvement plays an important role in the adoption of KC practices, either through the division of labor or by helping the mother to feel at ease [11]. For this reason, we support the idea of reformulating the concept of “Kangaroo Mother Care” or the “Kangaroo Mother Method” as “Kangaroo Care”, as suggested by Nyondo-Mipando et al. [6]. Rethinking this term makes it possible to expand the necessary scope for the upscaling of KM. In this low-resource setting, the aim would be to have an entire community involved in the sustainable practice of KC [6].

The healthcare facilities in which we conducted this research said that they integrate and apply KC for LBW infants. However, as in other studies, we observed inconsistencies between guidelines and practices, resulting in heterogeneity in the application of the method [29, 30]. The main challenges lie in the continuation of KC at home, as observed by Kinshella et al. [31] in Malawi, and the lack of follow-up. The practice of KC, as observed in families, is inconsistent with official WHO recommendations, according to which the infant should be carried continuously for more than 20 hours per day. This goal was far from being achieved in the households interviewed. Current practice in the field corresponds more to what has been described as “the intermittent practice of KC” [26, 32], as observed in Uganda [18]. All the women participating in this study expressed reluctance to practice KC continuously given their household chores and other work and perceptions surrounding the vulnerability of the child, which affected the application of KC practices at night. A systematic review revealed that intermittent KC has a negative impact on the growth of the child [33]. However, there is little evidence concerning the optimal duration of KC to achieve positive results. In addition to skin-to-skin contact, the correct positioning of the baby, an adequate duration of KC, exclusive breastfeeding, and follow-up are equally important for successful KC, and these fundamental objectives are not being met. The quality of KC implementation influences its effectiveness and outcome. The deviations in the implementation of KC observed here are highly disturbing, as these steps constitute the essential foundations of the practice of KC and such deviations can compromise the ability of the intervention to achieve the targeted objective, its scale up, as rightly pointed out by Mehjabeen et al. [10].

## Conclusions and recommendations

Previous studies have investigated the implementation of KC at hospital level in Madagascar, but feedback on the application of the practice in community settings in Madagascar is rare. This article is based on research carried out to identify the conditions for the success of KC, and obstacles to its implementation, at basic health centers, with the aim of guiding national recommendation protocols for the sustainable scaling up of KC. Our study highlights obstacles leading to incomplete and imprecise knowledge, and the heterogeneous practice of KC not in line with official WHO guidelines.

KC practices are globally accepted and generally in line with local cultural notions of the need to keep the child warm, but failings of the medical system undermine its application, given the lack of training of healthcare professionals, inadequate infrastructure, and a lack of monitoring. The scaling up of KC in Madagascar therefore remains a major challenge and, given current policies of the Ministry of Health, the implementation of KC in community settings is inadequate. (1) The integration of systematic training in KC into training modules for healthcare professionals (accompanied by follow-up), (2) the establishment of adequate infrastructures, and (3) the organization of follow-up with community agents will improve the practice of the method. However, these components must be accompanied by greater commitment from the Ministry of Health at national level (this is highlighted by the urgency of finalizing the KC curriculum and integrating it into the maternal and neonatal healthcare curriculum). If the ministry is unable to take charge of the transmission of these skills, we recommend that it provides (4) support to national associations, like Compassion Madagascar, to guarantee the sustainability and extension of training and professional development concerning KC. Strong and committed national associations working in unison with professional trainers facilitate the scaling up of KC, as observed in Finland, Ghana, India, Indonesia, South Africa, Sweden, Tanzania [7], and Madagascar, with Compassion Madagascar. KC is described as a low-cost solution adapted to contexts with limited resources, but it should be noted that it requires an investment in training, human resources, awareness, monitoring, and community engagement — basic needs that could also be financed by international aid agencies.

We suggest (5) further sensitization: during the prenatal period, with the identification of risk factors for preterm birth by healthcare and community workers, and during the postnatal period, through the promotion of KC and the benefits of family support, an adequate duration of KC, exclusive breastfeeding, and skin-to-skin contact. Awareness could be raised by the media (especially television and radio). In addition, community involvement, including that of men, and aiming to help the community take responsibility, would facilitate the adoption of KC. The role of community agents in Madagascar is fundamental for governing community mobilization and could serve as a means of support in the event of understaffing and overburdening with responsibilities, potentials obstacles to the adoption of KC described in Africa and Asia [24].

### Supplementary Information

**Additional file 1: Appendix A.** Interview guide for mothers of preterm infants who have practiced the kangaroo mother method. **Appendix B.** Interview guide for parents of prematurely born children who have practiced KMC and/or relatives who have practiced KMC. **Appendix C.** Interview guide for CSB caregivers. **Appendix D.** Focus group guide.

## Data Availability

The datasets used and/or analyzed during the current study available from the corresponding author on reasonable request.

## Footnotes

1 The community setting participates in health promotion and in the operation and management of basic health structures; it is deferred to the health district level and consists of the provision of services through community agents and advanced strategy health agents, basic health facilities for initial contacts and the user’s gateway to the health system: Basic Health Center (BHC).

2 Numbers of infants born with a LBW: 2590 in Analamanga in 2021 and 2047 in Boeny, according to the Family Health Directorate of the Public Health Ministry of Madagascar

3 Knowledge transfer is the set of processes by which the use of knowledge from research to orient actions and political decisions is promoted.

4 This article relates more specifically to infants who received KC home. We do not, therefore, have data concerning the follow-up of these preterm infants who should have received special care.
