# Supplementary material for: Anthropological overview of kangaroo care in community settings in Madagascar
Source: BMC Womens Health. 2023 Nov 23;23:623. doi: 10.1186/s12905-023-02781-7 (PMC10668420; doi:10.1186/s12905-023-02781-7)
Supplement: Supplementary file 1 — Additional file 1: Appendix A. Interview guide for mothers of preterm infants who have practiced the kangaroo mother method. Appendix B. Interview guide for parents of prematurely born children who have practiced KMC and/or relatives who have practiced KMC. Appendix C. Interview guide for CSB caregivers. Appendix D. Focus group guide. [file 12905_2023_2781_MOESM1_ESM.docx]

**Appendix A: INTERVIEW GUIDE FOR MOTHERS OF PRETERM INFANTS WHO HAVE PRACTICED**

**THE KANGAROO MOTHER METHOD**

1. **Identification**

| Name of respondent |  | | | | | | | | |
| --- | --- | --- | --- | --- | --- | --- | --- | --- | --- |
| Identification of the respondent |  | | | | | | | | |
| Age |  | | | | | | | | |
| Level of study |  | | | | | | | | |
| Professional activity |  | | | | | | | | |
| Marital status | - Single: - Divorced : - Widow : - Married: Age: Work: | | | | | | | | |
| Number of children | TOTAL :  BOY: age:  GIRL: age: | | | | | | | | |
| Month of birth of the premature child. | 1 | 2 | 3 | 4 | 5 | 6 | 7 | 8 | 9 |
|  |  |  |  |  |  |  |  |  |  |
| Age and birth weight of the child |  | | | | | | | | |
| Place of delivery: at home/CSB. |  | | | | | | | | |
| Direct debit |  | | | | | | | | |
| Fokontany |  | | | | | | | | |
| District |  | | | | | | | | |
| Municipality |  | | | | | | | | |
| Region |  | | | | | | | | |
| Province |  | | | | | | | | |

1. **Socio-professional issues**
   1. How old are you?
   2. What grade did you study in?
   3. What is your job?
   4. Are you married or not? if yes: how old is your husband? what is his job?
   5. How many children do you have? How old are they each?
   6. How many people are in your household?
2. **Pregnancy story**
3. How did your pregnancy go?
4. During your pregnancy, were there any significant events that occurred (to you, your family, your child) in your marital, professional, or other in your life?
5. How did you experience these events?
6. How often was your husband present during your pregnancy? And after the pregnancy?
7. **Perception of prematurity**
8. How do you explain your child's prematurity?

Points to underline if not mentioned in the answer:

Did you have any financial or material constraints? Were there any medical problems?

Have you had any prenatal consultations?

Are there any hereditary diseases, chronic illnesses, or a history of pregnancy?

In your family, were there already premature births? Who were they?

1. Did you receive information about prematurity during your pregnancy?
2. **Perception of the KMC**
3. How did you discover the KMC?
4. Who trained you to practice KMC?

Can you tell us about your first impression when you were told about adopting KMC for your baby? (Did it seem feasible? What was your motivation to do it? Did you feel any reluctance, if so, what were they?)

1. Did you feel sufficiently supervised to set up the KMC?
2. How did you feel during the practice of KMC?
3. Did you receive help during the portage? By whom?
4. Who does the cooking and cleaning in your home? (if the mother answers that it is her, how did she do it at the same time as the KMC?)
5. How was the KMC during the "mifana" confinement?
6. What have been the links to the practice of KMC?
7. What advice have you received for the practice of KMC at home?
8. How was the follow-up at home? Who did the follow-up?

23.Are there any women practicing KMC in your community?

24.What is your assessment of the KMC?

25.What do you think should be improved in the practice of KMC?

**Appendix B: INTERVIEW GUIDE FOR PARENTS OF PREMATURELY BORN CHILDREN**

**WHO HAVE PRACTICED KMC AND/OR RELATIVES WHO HAVE PRACTICED KMC**

1. **IDENTIFICATION**

| Name of respondent |  | | | | | | | | |
| --- | --- | --- | --- | --- | --- | --- | --- | --- | --- |
| Identification of the respondent |  | | | | | | | | |
| Age |  | | | | | | | | |
| Level of study |  | | | | | | | | |
| Professional activity |  | | | | | | | | |
| Marital status | - Single: - Divorced : - Widow(er) : - Married: Age: Work : | | | | | | | | |
| Number of children | TOTAL :  BOY: age:  GIRL: age: | | | | | | | | |
| Month of birth of the premature child. | 1 | 2 | 3 | 4 | 5 | 6 | 7 | 8 | 9 |
|  |  |  |  |  |  |  |  |  |  |
| Age of the child low weight |  | | | | | | | | |
| Place of delivery: at home/CSB. |  | | | | | | | | |
| Direct debit |  | | | | | | | | |
| Fokontany |  | | | | | | | | |
| District |  | | | | | | | | |
| Municipality |  | | | | | | | | |
| Region |  | | | | | | | | |
| Province |  | | | | | | | | |

1. How old are you?
2. What grade did you study in?
3. What is your job?
4. Are you married or not? if yes: how old is your wife? what is her occupation? / if it is a relative: add (what is your relationship with the mother who gave birth to a premature or low birth weight baby?
5. How many children do you have? How old are they each?
6. How many people live in your household?
7. PREGNANCY
8. How did your wife/daughter/sister/niece's pregnancy go? (Did any significant events occur? How do you think she experienced these events? Did you have any financial or material constraints? / Did she have any financial or material constraints?
9. For fathers: During pregnancy, what did you do? Can you describe a typical day?
10. PREMATURITY
11. How do you explain your child's prematurity? (or your grandchild, nephew/niece?) (Were there any medical problems? Did she have prenatal consultations? Are there any hereditary diseases, chronic illnesses, or a history of pregnancy? Was there a history of premature births in your family? )
12. Do you know if she received any information about prematurity during her

pregnancy?

1. KMC
2. How did you discover the KMC?
3. Have you been trained in the practice of KMC?
4. Can you tell us about your first impression when you were told about the adoption of KMC for the baby? (Were you reluctant? motivated?)
5. Have you been sufficiently supervised in the functioning of the KMC?
6. Did you feel involved in the practice of KMC?
7. Did you experience any difficulties in practicing KMC?
8. Did you receive any advice on returning home?
9. How was the follow-up at home?
10. What is your assessment of the KMC?
11. What do you think should be improved in the practice of KMC?

**Appendix C: INTERVIEW GUIDE FOR CSB CAREGIVERS**

1. **IDENTIFICATION**

| Name of respondent |  | |
| --- | --- | --- |
| Maintenance code |  | |
| Identification of the respondent |  | |
| Age |  | |
| Main activity |  | |
| Level of education |  | |
| Number of children | Boy: | age : |
|  |  |  |
|  | Girl: | Age: |
|  |  |  |
| Marital status | - Single - Divorced - Widow(er) : - Married: Age: Work : | |
| Home Follow-up Officer | Yes: No: | |

1. **Career path**
2. How old are you?
3. What is your function?
4. Can you tell us briefly about your professional background? *(where were you trained?)*
5. How long have you been here?
6. **PREMATURITY**
7. Why do you think women have premature babies?
8. How do they perceive the causes of prematurity?
9. During ANC, do you raise awareness of the risks of prematurity? *(If yes, how? If no, why?)*
10. **KMC**
11. Where were you trained in KMC (by whom, for how long, which key documents?)
12. Can you explain to us how a preterm delivery is carried out? What is the care provided here? (What materials? incubators?)
13. What is the management of a low birth weight child? and the management of a child born before term? What is the difference?
14. Can you explain to us the course of the KMC in your CSB?
15. What are the fees required to practice KMC?
16. In your opinion, what are the requirements and conditions to practice KMC in a community setting?
17. Who explains the course of the KMC to the parents of premature children?

How do they do it?

1. How do parents feel about adopting KMC for their child? Have you met any reluctant parents? How do you explain the reluctance and refusal of some parents?
2. Do you give advice for the continuation of the KMC at home?
3. How is the follow-up of the KMC at home?
4. Who does the follow-up? Have you considered CHWs as partners in the follow-up of the KMC at home?
5. What difficulties do you feel about the practice of KMC in your CSB?
6. In your opinion, what elements should be taken into account for the good functioning of the KMC in community settings?
7. Are there any caregivers who are reluctant to use KMC? If so, why?

**Appendix D: FOCUS GROUP GUIDE**

1. **IDENTIFICATION**

Code focus group

Date: ...............................................

Moderator's name:

Name of the observer:

Number of participants:

| N° | Full name | gender | Age | STATUS MATR | NUMBER OF CHILDREN | SIGNATURE |
| --- | --- | --- | --- | --- | --- | --- |
|  |  |  |  |  |  |  |
|  |  |  |  |  |  |  |
|  |  |  |  |  |  |  |
|  |  |  |  |  |  |  |
|  |  |  |  |  |  |  |

The addition of the focus group is intended to deepen and support certain information and analyses from the individual interviews (to be developed following the individual interviews).

1. **The perception of KMC (e.g. social norms and family involvement in the practice of KMC);**
2. **The transmission of information by health care personnel.**
3. **Expectations for the effectiveness of the KMC and expectations for improving the KMC**
